# Long-Term Mortality and Quality of Life Among Adults With Mild Traumatic Brain Injury

**DOI:** 10.1001/jamanetworkopen.2026.29041

**Published:** 2026-08-13

**Authors:** Kantesh Kumar, Komal Abdul Rahim, Mehrose Ahmad, Rashid Ali, Sheza Hassan, Tanweer Ahmed, Saima Mushtaq, Muhammad Shahzad Shamim, Zaheer Babar, Yasir Shafiq, Huba Atiq, Adil Haider, Junaid Razzak

**Affiliations:** 1 Centre of Excellence for Trauma and Emergencies, The Aga Khan University, Karachi, Pakistan; 2 Weill Cornell Medicine, New York; 3 Department of Emergency Medicine, Weill Cornell Medicine, New York; 4 Department of Neurosurgery, Jinnah Postgraduate Medical Centre, Karachi, Pakistan; 5 Department of Emergency Medicine, Jinnah Postgraduate Medical Centre, Karachi, Pakistan; 6 Department of Surgery, The Aga Khan University, Karachi, Pakistan

## Abstract

**Importance:**

Mild traumatic brain injuries (mTBIs) are the most common form of TBI and are responsible for 1.37 million years lived with disability annually, disproportionately affecting low- and middle-income countries (LMICs), as 90% of TBIs occur in LMICs.

**Objective:**

To measure the long-term mortality (12 months), quality of life (QoL), and functional status of patients with mTBI in Pakistan.

**Design, Setting, and Participants:**

This cohort study was an analysis of prospectively collected data of patients with mTBI from a trauma registry of all injured patients from 2 tertiary care centers in Karachi, Pakistan, from December 2021 to May 2024, who were followed up for 12 months. Participants were adult patients (aged >18 years) admitted with mTBI and enrolled in the registry with recorded outcomes at 1, 3, 6, and 12 months.

**Exposure:**

Mild TBI (Glasgow Coma Scale [GCS] score of 13-15).

**Main Outcomes and Measures:**

Long-term mortality (12-month cumulative and follow-up) was the primary outcome, and QoL was the secondary outcome.

**Results:**

A total of 602 patients presented with mTBI. Most were aged 18 to 39 years (336 patients [55.8%]), were male (502 patients [83.4%]), and experienced mTBIs from road traffic collisions (427 patients [71.1%]), or falls (130 patients [21.6%]). Surgical interventions were performed in 176 cases (29.2%) and were associated with 51% lower odds of 1-month cumulative mortality (adjusted hazard ratio, 0.44; 95% CI, 0.21-0.87). The inpatient, cumulative 1-month, and 12-month mortality rates were 4.6%, 8.9%, and 13.6%, respectively. Patients with a GCS score of 13 (20.2%) and 14 (20.1%) had a higher 12-month mortality rate as compared with patients with a GCS score of 15 (8.3%). Of 345 patients with follow-up data at 1 year, 269 (78.0%) achieved complete independence by 1 year. The functional engagement domain showed the highest rate of recovery (318 patients [92.2%]), while the physical well-being and recovery domain had the lowest, with 286 (82.9%) experiencing disability, particularly those with a GCS score of 13 and 14; only 59 (17.1%) reported no physical disability at 12 months.

**Conclusion and relevance:**

In this cohort study of patients with mTBI in Pakistan, 12-month mortality was approximately 2-fold higher in patients with a GCS score of 13 to 14 (20.2% and 20.1%) compared with a GCS score of 15 (8.3%), alongside persistently poorer quality of life in lower-GCS score groups throughout the recovery period.

## Key Points

**Question:**

What are the long-term mortality, quality of life (QoL), and functional outcomes of patients with mild traumatic brain injury (mTBI) in a low- and middle-income country (LMIC)?

**Findings:**

This cohort study including 602 patients found a 12-month cumulative mortality of 13.6% among admitted patients with mTBI (20% for Glasgow Coma Scale [GCS] score of 13-14 and 8% for GCS score of 15). By 12 months, 92.2% of patients recovered functionally; 17.1% recovered physically.

**Meaning:**

These findings suggest both the necessity of structured postdischarge follow-up for high-risk patients and the limitations of existing mTBI classifications in capturing true injury severity within LMIC contexts.

## Teaser

This cohort study evaluates the long-term mortality, quality of life, and functional status of patients with mild traumatic brain injury (mTBI) in Pakistan.

## Introduction

Traumatic brain injuries (TBIs) alter brain function or cause brain pathology due to external force trauma. Globally, over 69 million people experience a TBI annually, resulting in greater than 8.1 million years of living with disability.^1,2^ Given their widespread occurrence and severe clinical consequences, TBIs are now widely recognized as a significant global public health crisis.^3^

TBIs are categorized as either mild, moderate, or severe based on a patient’s Glasgow Coma Scale (GCS) score at presentation to a health facility. Of the 3 types, mild TBIs (mTBIs) are the most common globally (60%-95%), and cause significant morbidity. mTBIs may present with diverse symptoms, such as headaches, dizziness, nausea, fatigue, visual impairment, insomnia, attentional deficits, and memory problems.^3,4,5,6,7,8,9^ These symptoms can fluctuate in both intensity and duration, with the potential to significantly affect a patient’s long-term quality of life (QoL).

Although TBIs are prevalent globally, current data suggest that their prevalence may be 3 to 4 times greater in lower- and middle-income countries (LMICs) than in high-income countries (HICs).^10^ Furthermore, patients experiencing mTBIs in LMICs also have an increased degree of morbidity compared with patients in HICs.^1,10,11^ One study^12^ conducted in the US revealed a 5-year follow-up mortality rate of 7% after an mTBI. However, despite the growing prevalence of mTBIs in LMIC settings, there is a scarcity of high-quality studies on their epidemiology, management, and outcomes.^1,13^

Considering the long-term impact that mTBIs may impose, coupled with their social and financial consequences on both an individual and their collective society, further investigations on mTBIs are needed. This is especially important in the context of LMICs, where individuals are not only more prone to experiencing mTBIs, but are also less likely to receive adequate and timely care following such incidents.^14^ LMICs often witness infrastructural and resource issues, coupled with uncoordinated, informal, and ineffective prehospital and ambulance services.^15,16^ One meta-analysis^10^ on TBIs in LMICs reported a pooled mortality rate of 4.33% for mTBIs. However, there is a dearth of literature on the long-term outcomes after mTBI in LMICs. To fill this gap, this study aims to measure long-term mortality, QoL, and functional status for patients with mTBI in Pakistan.

## Methods

### Study Design and Setting

We conducted a prospective cohort study using data spanning from December 2021 to May 2024 within a multicenter trauma registry from 2 large centers in Karachi, Pakistan. The registry enrolled all adult patients (aged ≥18 years) admitted to these 2 centers in inpatient units. Details on the registry have been published previously.^17^ The study was approved by the ethical review committee of the Aga Khan University (reference No. 2021-6251-18973). This study was conducted and reported in accordance with Strengthening the Reporting of Observational Studies in Epidemiology (STROBE) reporting guideline for cohort studies. Written informed consent was obtained by the trained data collectors during the inpatient data collection. During that process, participants were also informed regarding follow-up data collection at 1, 3, 6, and 12 months. Verbal consent was obtained during the follow-up calls for data collection.

### Population of Interest

All patients diagnosed with mTBIs and enrolled in the Trauma Registry with recorded outcomes at 1, 3, 6, and 12 months were included in this study. mTBIs were defined using the GCS, including patients with a reported GCS score of 13 to 15. Patients with tracheostomies and arrival GCS scores recorded out of 10 (verbal component of GCS scores not recorded) were excluded as they could not be reliably classified as mild. The quality check was confirmed on the dead patients via retrospective file review to ensure the inclusion of only patients with mTBI. Following the data’s quality check, 602 of 610 patients (98.6%) were confirmed to have mild TBI on hospital arrival. eFigure 1 in Supplement 1 demonstrates a schematic of the inclusion and exclusion flow of patients.

### Variables of Interest

The primary outcome was inpatient mortality and postdischarge mortality, defined as the death of a patient during their hospital stay and after discharge. Secondary outcomes included disability, determined through the functional independence measure (FIM) with a score ranging from 18 to 126 (with a higher score indicating greater independence), and quality of life assessed through revised-trauma QoL (RT-QoL).^18^

Sociodemographic variables captured were age (18-39, 40-59, and ≥60 and years), gender (male or female), and educational status (no formal education, primary education, secondary education, intermediate, or graduation and above). Injury details included the mechanism of injury (road traffic crashes [RTCs], falls, assault, and others), and injury severity (mild, moderate, severe, and profound). Clinical variables included the GCS scores of patients (13, 14, or 15), the service under which the patient was admitted (ward, high-dependency unit, intensive care unit, or operation theaters), and TBI diagnosis (contusions, epidural hematoma, subdural hematoma, skull fracture, facial fractures, intracerebral hemorrhage, and vertebral fracture).

Discharge outcomes were captured as: discharged home or to another facility, died, left against medical advice, or unknown. The cumulative mortality of patients at 12 months was estimated using the Turnbull nonparametric maximum likelihood estimator (NPMLE) with interval censoring.

### Statistical Analysis

Descriptive analysis was conducted using frequencies and percentages for categorical variables. Interval-censored survival analysis estimated unadjusted and adjusted hazard ratios (HRs) with 95% CIs for 12-month mortality, with time 0 at injury and right-censored observations assigned the time from injury to last known alive visit. A semiparametric proportional hazards model was fitted with the following prespecified covariates: age category, sex, mechanism of injury, injury severity score (ISS) category, comorbidities, GCS score, and surgical intervention. Unadjusted HRs were estimated from separate univariate models. Nonparametric cumulative mortality curves were estimated using the Turnbull NPMLE, stratified by GCS score (579 patients; records with missing injury dates or inconsistent date bounds excluded) (eFigure 2 in Supplement 1). A sensitivity analysis assessed the loss to follow-up by varying only genuinely lost patients (those without a confirmed status at the final visit); patients confirmed alive at the 12-month visit were treated as noninformative censoring and left unchanged. Genuinely lost patients were assumed alive to the end of intended follow-up (best case; last known alive time extended to 12 months) or dead at the last known contact (worst case).

RT-QoL responses were collapsed into 3 categories (agree, disagree, or no opinion). Within each domain, symptom burden was quantified by summing items for which patients endorsed the adverse response, categorized as no symptoms (0), mild (1-2), moderate (3-4), and severe (5-6). To examine QoL trajectories over time and by GCS score, ordinal cumulative-link mixed-effects models were fitted separately for each domain with time, GCS score, and their interaction as the primary variables, adjusted for age, sex, ISS category, surgical intervention, comorbidities, and FIM at discharge, with a random intercept for the patient. Patients who died before a given time point were excluded from QoL analysis at that and subsequent time points.

We assessed missing data patterns across all FIM and RT-QoL items at each follow-up, and identified nonrandom missingness (missing completely at random [MCAR] rejected), with variables indicating a missing at random (MAR) mechanism. The Little MCAR test was rejected (*P* < .05), and logistic regression models confirmed MAR mechanism. Multiple imputation was performed using multiple imputation by chained equations with predictive mean matching (20 datasets, 30 iterations), including QoL items, FIM scores (except 12-month time point), baseline covariates, and follow-up status indicators. Outcomes for deceased patients and individuals with no observation data at any time point were not imputed by design. Item-level QoL imputations were aggregated into domain scores only when at least 1 item was observed or imputed, with fully missing domains retained as unknown. Imputed estimates were pooled using the Rubin rule. QoL mixed-effects model results were pooled using a manual implementation of the Rubin rules, as clmm is not natively supported by standard pooling functions.

All analyses were carried out using R version 4.4.1 (R Project for Statistical Computing). A 2-sided *P* < .05 was considered statistically significant.

## Results

A total of 602 patients presented with mTBI, with 336 (55.8%) aged 18 to 39 years. A total of 502 were male (83.39%), 170 received no formal education (28.91%), and most mTBIs resulted from RTCs (427 patients [71.05%]), followed by falls (130 patients [21.6%]). Older patients (aged ≥60 years) more commonly presented with a GCS score of 13 (25 patients [17.73%]) or 14 (33 patients [22.92%]) than 15 (40 patients [12.62%]). Within the GCS 13 group, RTCs were the most common mechanism (108 patients [76.60%]), followed by falls (16 patients [18.44%]), assault (5 patients [3.55%]), and other causes (2 patients [1.42%]). A total of 176 patients (29.2%) received surgical intervention, most commonly craniotomies (61 patients [43.26%]), followed by burr holes (23 patients [16.31%]) and craniectomies (22 patients [15.60%]). The median (IQR) length of stay was longest in the GCS 13 group (2.28 [1.25-4.34] days), followed by GCS scores of 14 (2.01 [1.14-4.02] days) and GCS scores of 15 (1.90 [1.02-3.57] days) (Table 1). eTable 1 in Supplement 1 shows the demographic case mix of patients by the type of procedures. Burr holes were most common in patients aged 60 years and older (52.17%), whereas craniectomies were evenly distributed between the 40 to 59 and 60 years or older age groups (36.36% each).

**Table 1.** Distribution of Epidemiological and Clinical Characteristics Among Patients With Mild Traumatic Brain Injury (TBI)

Characteristic	Participants, No. (%)				*P* value
	Total (N = 602)	GCS 13 (n = 141)	GCS 14 (n = 144)	GCS 15 (n = 317)	
Age group, y					
18-39	336 (55.81)	77 (54.61)	76 (52.78)	183 (57.73)	.09
40-59	168 (27.91)	39 (27.66)	35 (24.31)	94 (29.65)	
≥60	98 (16.28)	25 (17.73)	33 (22.92)	40 (12.62)	
Gender					
Male	502 (83.39)	118 (83.69)	122 (84.72)	262 (82.65)	.90
Female	100 (16.61)	23 (16.31)	22 (15.28)	55 (17.35)	
Educational status					
No formal education	170 (28.91)	46 (33.33)	46 (32.62)	78 (25.24)	.06
Primary education	126 (21.43)	33 (23.91)	28 (19.86)	65 (21.04)	
Secondary education	144 (24.49)	30 (21.74)	43 (30.50)	71 (22.98)	
Intermediate	68 (11.56)	13 (9.42)	10 (7.09)	45 (14.56)	
≥Graduation from university	80 (13.61)	16 (11.59)	14 (9.93)	50 (16.18)	
Missing	14	3	3	8	
Mechanism of injury					
RTCs	427 (71.05)	108 (76.60)	99 (68.75)	220 (69.62)	.09
Fall	130 (21.63)	26 (18.44)	40 (27.78)	64 (20.25)	
Assault	29 (4.83)	5 (3.55)	3 (2.08)	21 (6.65)	
Others	15 (2.50)	2 (1.42)	2 (1.39)	11 (3.48)	
Missing	1	0	0	1	
Role in RTC (RTC = 427)					
Passenger	47 (11.22)	6 (5.77)	4 (4.12)	37 (16.97)	NA
Pillion passenger	47 (11.22)	17 (16.35)	12 (12.37)	18 (8.26)	
Driver	12 (2.86)	2 (1.92)	1 (1.03)	9 (4.13)	
Riders	249 (59.43)	65 (62.50)	59 (60.82)	125 (57.34)	
Pedestrian	64 (15.27)	14 (13.46)	21 (21.65)	29 (13.30)	
Missing	8	4	2	2	
Primary vehicle (RTC = 427)					
Motorbike	323 (76.36)	88 (83.02)	76 (77.55)	159 (72.60)	NA
Car	25 (5.91)	3 (2.83)	2 (2.04)	20 (9.13)	
Rickshaw	8 (1.89)	0	1 (1.02)	7 (3.20)	
Bus	6 (1.42)	1 (0.94)	0	5 (2.28)	
Pedestrian	50 (11.82)	8 (7.55)	18 (18.37)	24 (10.96)	
Other	11 (2.60)	6 (5.66)	1 (1.02)	4 (1.83)	
Unknown	4	2	1	1	
ISS category					
Mild (≤8)	54 (8.97)	8 (5.67)	7 (4.86)	39 (12.30)	.14
Moderate (9-15)	326 (54.15)	77 (54.61)	84 (58.33)	165 (52.05)	
Severe (16-24)	188 (31.23)	48 (34.04)	44 (30.56)	96 (30.28)	
Profound (≥25)	34 (5.65)	8 (5.67)	9 (6.25)	17 (5.36)	
Services					
Ward	495 (83.05)	127 (91.37)	130 (91.55)	238 (75.56)	<.001
HDU	11 (1.85)	4 (2.88)	1 (0.70)	6 (1.90)	
ICU	90 (15.10)	8 (5.76)	11 (7.75)	71 (22.54)	
Missing	6	2	2	2	
Surgical intervention					
Yes	176 (29.24)	21 (14.89)	34 (23.61)	121 (38.17)	<.001
No	426 (70.76)	120 (85.11)	110 (76.39)	196 (61.83)	
Procedure type					
No	426 (70.76)	120 (85.11)	110 (76.39)	196 (61.83)	<.001
Cranial	124 (20.60)	16 (11.35)	30 (20.83)	78 (24.61)	
Noncranial	46 (7.64)	3 (2.13)	3 (2.08)	40 (12.62)	
Both	6(1.00)	2 (1.42)	1 (0.69)	3 (0.95)	
Procedure (n = 176)					
Craniotomy	61 (43.26)	12 (60)	16 (48.48)	33 (37.50)	NA
Craniectomy	22 (15.60)	2 (10)	6 (18.18)	14 (15.91)	
Burr hole	23 (16.31)	3 (15)	5 (15.15)	15 (17.05)	
Elevation of depressed fracture	14 (9.93)	1 (5)	3 (9.09)	10 (11.36)	
Laceration repair	11 (7.80)	1 (5)	1 (3.03)	9 (10.23)	
Other	10 (7.09)	1 (5)	2 (6.06)	7 (7.95)	
Missing	35	1	1	33	
TBI diagnosis					
Contusions	200 (33.2)	71 (50.35)	64 (44.44)	65 (20.50)	NA
Epidural hematoma	193 (32.1)	42 (29.79)	48 (33.33)	103 (32.49)	
Subdural hematoma	147 (24.4)	40 (28.37)	35 (24.31)	72 (22.71)	
Skull fracture	93 (15.4)	19 (13.48)	19 (13.19)	55 (17.35)	
Facial fracture	33 (5.5)	6 (4.26)	8 (5.56)	19 (5.99)	
Intracerebral hemorrhage	21 (3.5)	8 (5.67)	6 (4.17)	7 (2.21)	
Pneumocranium	19 (3.2)	4 (2.84)	7 (4.86)	8 (2.52)	
Vertebral fracture	14 (2.3)	NA	NA	14 (4.42)	
CT scan					
No	20 (3.32)	NA	NA	20 (6.29)	<.001
Yes	582 (96.68)	141 (100)	144 (100)	297 (93.69)	
MRI					
No	589 (97.84)	140 (98.29)	142 (98.61)	307 (96.85)	.30
Yes	13 (2.16)	1 (0.71)	2 (1.39)	10 (3.15)	
Length of stay (n = 565)	2 (1.10-3.89)	2.28 (1.25-4.34)	2.01 (1.14-4.02)	1.90 (1.02-3.57)	.08
Missing	NA	9	8	27	NA

Abbreviations: CT, computed tomography; GCS, Glasgow Coma Score; HDU, high-dependency unit; ICU, intensive care unit; ISS, injury severity score, MRI, magnetic resonance imaging; NA, not applicable; RTC, road traffic crash.

The differences between patients followed up and those lost to follow-up are described in eTable 2 in Supplement 1. There was a statistically significant difference in education (no formal education: 106 patients [25.5%] vs 64 [37.0%]), admission service (high-dependency unit; 333 patients [80.2%] vs 162 [89.5%]), and median (IQR) length of stay (2.11 [1.17-4.23] days vs 1.79 [0.98-3.04] days).

Table 2 reports patient outcomes at discharge and follow-up intervals stratified by GCS scores. The inpatient, cumulative 1-month, and 12-month mortality rates were 4.6%, 8.9%, and 13.6%, respectively. Discharge home rates were highest in patients with a GCS score of 15 (282 patients [94.63%]). The 12-month cumulative mortality (579 patients) is shown in eFigure 2 in Supplement 1. The overall cumulative 12-month mortality rate was 13.6%, with a rate of 20.2% and 20.1% for those with a GCS score of 13 and 14, as compared with 8.3% of patients with a GCS score of 15. The highest number of patients died 1 month after discharge compared with inpatient as well as follow-up intervals at which patient status was recorded (29 patients [6.21% of those with known 1-month status]). eTable 3 in Supplement 1 reports cumulative mortality under primary, best-case, and worst-case lost-to-follow-up (LTFU) assumptions. At 12 months, overall cumulative mortality was 13.6% (primary), with bounds of 12.3% (best case, LTFU assumed alive) and 40.4% (worst case, LTFU assumed dead). Patients with a GCS score of 15 had the lowest mortality under primary analysis (3.3%-8.3% at 1-12 months), while those with a GCS score of 13 and 14 had notably higher rates (18.6%-20.2% and 12.9%-20.1%, respectively).

**Table 2.** Patient Outcomes at Discharge and Follow-up

Characteristics	Participants, No. (%)			
	Total (N = 602)	GCS 13 (n = 141)	GCS 14 (n = 144)	GCS 15 (n = 317)
Discharge disposition				
Discharged to home	523 (92.08)	119 (88.81)	122 (89.71)	282 (94.63)
Discharged to other facility	2 (0.35)	NA	NA	2 (0.67)
Dead	26 (4.58)	11 (8.21)	8 (5.88)	7 (2.35)
Left against medical advice	17 (2.99)	4 (2.99)	6 (4.41)	7 (2.35)
Unknown	34	7	8	19
1 mo				
Alive	412 (88.22)	86 (78.18)	91 (83.49)	235 (94.76)
Dead	29 (6.21)	13 (11.82)	10 (9.17)	6 (2.42)
Not eligible^a^	26 (5.57)	11 (10.00)	8 (7.34)	7 (2.82)
Loss to follow-up	135	31	35	69
3 mo				
Alive	391 (85.37)	85 (77.27)	84 (79.25)	222 (91.74)
Dead	12 (2.62)	1 (0.91)	4 (3.77)	7 (2.89)
Not eligible^a^	55 (12.01)	24 (21.82)	18 (16.98)	13 (5.37)
Loss to follow-up	144	31	38	75
6 mo				
Alive	376 (84.30)	84 (77.06)	77 (76.24)	215 (91.10)
Dead	3 (0.67)	NA	2 (1.98)	1 (0.42)
Not eligible^a^	67 (15.02)	25 (22.94)	22 (21.78)	20 (8.47)
Loss to follow-up	156	32	43	81
12 mo				
Alive	347 (82.42)	76 (75.25)	75 (75.00)	196 (89.09)
Dead	4 (0.95)	NA	1 (1.00)	3 (1.36)
Not eligible^a^	70 (16.63)	25 (24.75)	24 (24.00)	21 (9.55)
Loss to follow-up	181	40	44	97
Cumulative mortality (n = 579), %^b^				
1 mo	8.9	18.6	12.9	3.3
3 mo	12.2	20.2	17.7	5.6
6 mo	13.1	20.2	20.1	7.3
12 mo	13.6	20.2	20.1	8.3
Follow-up mortality, No./total No. (%)^c^				
1 mo	29/441 (6.58)	13/99 (13.13)	10/101 (9.90)	6/241 (2.49)
3 mo	41/432 (9.49)	14/99 (14.14)	14/98 (14.29)	13/235 (5.53)
6 mo	44/420 (10.48)	14/98 (14.29)	16/93 (17.20)	14/229 (6.11)
12 mo	48/395 (12.15)	14/90 (15.56)	17/92 (18.48)	17/213 (7.98)

Abbreviations: GCS, Glasgow Coma Scale; NA, not applicable.

^a^ Patients who died at the previous follow-up are represented as not eligible.

^b^ Cumulative mortality: Turnbull NPMLE interval-censored estimate from injury, accounting for censoring.

^c^ Follow-up mortality: crude conditional proportion of deaths among patients with known status at that visit (deaths or known-status denominator).

Table 3 presents the unadjusted and adjusted hazard ratios, along with 95% CIs, for 12-month mortality from the interval-censored proportional hazards model. Being aged 60 years or older was associated with higher mortality in unadjusted (HR, 4.25; 95% CI, 2.42-7.47) and adjusted analysis (aHR, 2.98; 95% CI, 1.44-6.13) compared with age 18 to 39 years. A GCS score of 13 (aHR, 1.96; 95% CI, 1.06-3.64) or 14 (aHR, 2.28; 95% CI, 1.23-4.22) was associated with significantly higher mortality as compared with a GCS score of 15. Patients who underwent surgical intervention (176 patients [29.2%]) had 51% lower odds of mortality (aHR, 0.49; 95% CI, 0.26-0.94) after adjustment for other factors.

**Table 3.** Factors Associated With Patient Outcome at 12-Month Follow-Up Using Univariable and Multivariable Interval-Censored Proportional Hazards Regression^a^

Characteristics	Participants, No. (%)		HR (95% CI)	
	Alive (n = 347)	Dead (n = 74)	Unadjusted	Adjusted
Age category, y				
18-39	196 (87.9)	27 (12.1)	1 [Reference]	1 [Reference]
40-59	109 (85.2)	19 (14.8)	1.40 (0.77-2.57)	1.08 (0.53-2.21)
>60	42 (60.0)	28 (40.0)	4.25 (2.42-7.47)	2.98 (1.44-6.13)
Gender				
Male	294 (84.2)	55 (15.8)	1 [Reference]	1 [Reference]
Female	53 (73.6)	19 (26.4)	1.88 (1.09-3.24)	1.77 (0.98-3.19)
Trauma				
RTA	278 (84.5)	51 (15.5)	1 [Reference]	1 [Reference]
Fall	69 (75.0)	23 (25.0)	1.66 (1.00-2.76)	1.33 (0.74-2.39)
ISS category				
Mild	38 (90.5)	4 (9.5)	1 [Reference]	1 [Reference]
Moderate	181 (83.4)	36 (16.6)	1.69 (0.06-46.93)	1.59 (0.06-40.85)
Severe	128 (79.0)	34 (21.0)	2.15 (0.08-60.10)	2.20 (0.08-56.82)
Comorbidities				
No	269 (85.4)	46 (14.6)	1 [Reference]	1 [Reference]
Yes	78 (73.6)	28 (26.4)	2.07 (1.27-3.36)	1.46 (0.79-2.68)
GCS				
15	196 (89.1)	24 (10.9)	1 [Reference]	1 [Reference]
14	76 (75.2)	25 (24.8)	2.66 (1.46-4.83)	2.28 (1.23-4.22)
13	75 (75.0)	25 (25.0)	2.45 (1.36-4.41)	1.96 (1.06-3.64)
Surgical interventions				
No	235 (79.9)	59 (20.1)	1 [Reference]	1 [Reference]
Yes	112 (88.2)	15 (11.8)	0.53 (0.28-1.01)	0.49 (0.26-0.94)

Abbreviations: GCS, Glasgow Coma Scale; HR, hazard ratio; ISS, injury severity score.

^a^ic_sp semiparametric proportional hazards. Time 0 = injury date. Bootstrap SE: 1000 samples, seed 2025. Likelihood-ratio test for GCS: χ^2^_2_ = 15.348; *P* ≤ .001. Five patients were excluded due to inconsistent dates.

Table 4 shows the quality-of-life trajectory. Physical well-being improved over time in patients who reported severe disability (86 patients [19.6%] at 1 month and 9 patients [2.6%] at 12 months), with patients shifting from the moderate and severe categories into the no and mild disability categories. Patients reported no disability in the functional engagement domain at 12 months (318 patients [92.2%]). The physical well-being and recovery domain had the lowest recovery, with 286 (82.9%) experiencing disability, particularly in those with a GCS score of 13 and 14. Emotional well-being improved at 12 months in patients who reported severe disability at 1 month (2 patients [0.6%] vs 31 [7.1%]). eTable 4 in Supplement 1 demonstrates that all quality of life trajectories stratified by GCS score over time at 1-, 3-, 6-, and 12-month follow-up.

**Table 4.** Distribution of Quality-of-Life Domains in Patients at 1-, 3-, 6-, and 12-Month Follow-Up

Characteristics	Participants, No. (%)			
	1 mo (n = 462)	3 mo (n = 462)	6 mo (n = 462)	12 mo (n = 462)
Physical well-being and recovery				
No disability	72 (16.4)	127 (30.1)	95 (22.8)	59 (17.1)
Mild disability	134 (30.5)	154 (36.5)	193 (46.3)	212 (61.4)
Moderate disability	147 (33.5)	95 (22.5)	109 (26.1)	65 (18.8)
Severe disability	86 (19.6)	46 (10.9)	20 (4.8)	9 (2.6)
Unknown	23	40	45	117
Functional engagement				
No disability	325 (74.0)	348 (82.5)	381 (91.4)	318 (92.2)
Mild disability	30 (6.8)	22 (5.2)	19 (4.6)	13 (3.8)
Moderate disability	22 (5.0)	13 (3.1)	4 (1.0)	6 (1.7)
Severe disability	62 (14.1)	39 (9.2)	13 (3.1)	8 (2.3)
Missing	23	40	45	117
Emotional well-being				
No disability	189 (43.1)	253 (60.0)	309 (74.3)	249 (72.2)
Mild disability	136 (31.0)	104 (24.6)	73 (17.5)	68 (19.7)
Moderate disability	83 (18.9)	44 (10.4)	26 (6.2)	26 (7.5)
Severe disability	31 (7.1)	21 (5.0)	8 (1.9)	2 (0.6)
Missing	23	40	46	117

Table 5 reports factors associated with the trajectory of recovery for each domain of quality of life over time. All domains improved significantly over 12 months, with the most recovery in functional engagement. Patients with a GCS score of 13 and 14 had significantly higher odds of worse emotional well-being compared with those with a GCS score of 15 (odds ratio [OR], 2.11; 95% CI, 1.19-3.72 and OR, 1.84; 95%, CI 1.07-3.16, respectively). The time and GCS score of 13 interaction was significant for functional engagement (OR, 0.86; 95% CI, 0.74-0.99), suggesting faster recovery compared with patients with a GCS score of 15. Being aged older than 60 and surgical intervention were associated with worse functional engagement and emotional well-being, respectively.

**Table 5.** Ordinal Mixed-Effects Model of Quality-of-Life Outcomes Over 12 Months Following Mild Traumatic Brain Injury

Variable	OR (95% CI)^a^		
	Emotional well-being	Functional engagement	Physical well-being
Term			
Time (months)	0.85 (0.81-0.89)	0.83 (0.78-0.89)	0.93 (0.90-0.96)
GCS score (reference group: 15)			
13	2.11 (1.19-3.72)	1.16 (0.53-2.53)	0.79 (0.49-1.28)
14	1.84 (1.07-3.16)	1.36 (0.65-2.84)	1.49 (0.95-2.34)
Age category (reference group: 18-39 y)			
40-59 y	1.15 (0.74-1.79)	1.42 (0.76-2.69)	1.38 (0.99-1.92)
≥60 y	1.50 (0.87-2.61)	4.50 (2.04-9.92)	1.90 (1.22-2.94)
Gender (reference group: male)			
Female	1.33 (0.85-2.08)	1.01 (0.52-1.95)	1.03 (0.72-1.46)
ISS category (reference group: mild)^b^			
Moderate	1.68 (0.94-3.02)	0.98 (0.46-2.08)	1.42 (0.93-2.15)
Severe	1.18 (0.62-2.23)	0.69 (0.29-1.64)	1.10 (0.70-1.74)
Profound	1.07 (0.49-2.38)	1.99 (0.66-6.02)	1.62 (0.87-3.01)
Surgery (reference group: no)			
Yes	1.78 (1.21-2.63)	1.06 (0.61-1.85)	0.97 (0.73-1.30)
Comorbidities (reference group: no)			
Yes	1.38 (0.86-2.22)	1.46 (0.75-2.83)	1.36 (0.94-1.96)
FIM at discharge	0.99 (0.98-1.00)	0.96 (0.95-0.97)	0.98 (0.98-0.99)
Time × GCS 13^c^	0.98 (0.91-1.06)	0.86 (0.74-0.99)	0.99 (0.94-1.06)
Time × GCS 14^c^	1.02 (0.94-1.10)	0.96 (0.85-1.08)	0.98 (0.93-1.04)

Abbreviations: GCS, Glasgow Coma Score; ISS, injury severity score; FIM, functional independence measure; OR, odds ratio.

^a^ ORs greater than 1 indicate higher cumulative odds of a worse disability category. The proportional odds assumption applies across all category boundaries.

^b^ Outcome coded as a 4-level ordinal scale: no symptoms, mild, moderate, and severe.

^c^ Time × GCS interaction terms test whether GCS groups diverge or converge in quality of life trajectory over the 12-month period.

The FIM categorization is outlined in eTable 5 in Supplement 1. At admission, 37 patients reported modified to complete independence, with most having a GCS score of 15 (33 patients). Among patients with a GCS score of 13, 55 (62.5%) required minimal to maximal assistance at discharge. By 12 months, 269 patients (78.0%) had achieved complete independence. At the end of the study, 59 (17.1%), 249 (72.2%), and 318 (92.2%) patients reported no disability in physical or emotional well-being or functional engagement, respectively.

## Discussion

Our study measured inpatient and 12-month cumulative mortality in patients with mTBI. The cumulative mortality rate was 4.6% and 13.6% during the hospital stay and 12 months after discharge, respectively. Around 29.2% of patients had surgical interventions, which were significantly associated with 51% lower odds of mortality. Over time, patient QoL improved significantly, with 72% and 92% denying any disability in emotional well-being and functional engagement 12 months after discharge, respectively.

Our study reported a 12-month cumulative mortality rate of 13.6% for mTBIs. There is a dearth of literature on mTBI long-term mortality in LMICs, which might be attributed to a lack of standard controls, selection bias, and loss of follow-up.^19^ However, some literature is available on overall TBI cohorts; one study^20^ conducted in Uganda for patients with TBI with a median follow-up of 18.6 months revealed a follow-up mortality rate of 9%, compared with 13.6% in our study. Our study’s higher postdischarge mortality may be due to misclassification of patients as having an mTBI. The highest mortality was observed in those with a GCS score of 13 (20.2%) and 14 (20.1%), compared with a mortality rate of 8.3% for patients with a GCS score of 15. This pattern may reflect the well-documented limitations of GCS score as a sole classifier of injury severity; GCS scores can be artificially lowered in intubated or sedated patients, and GCS scores of 13 in particular have been shown to align more closely with moderate TBI outcomes in terms of pathology and prognosis than its mild designation implies. Consequently, some of the observed mortality may represent undertriage of moderate-to-severe injuries rather than true mTBI outcomes. Premature hospital discharge may contribute to this finding, as patients had a median length of stay of just 2 days in our study.

Our study’s reported inpatient mortality of 4.58% is similar to the 4% pooled mortality rate in patients with mTBI reported in a meta-epidemiological study^10^ of TBI studies from LMICs. The reported inpatient mortality rate for mTBI varies in individual LMIC studies; one study^21^ conducted in India reported a mortality rate of 0.6%, while another study in Uganda reported a mortality rate of 4.7% in patients with mTBI. These mortality rates from LMICs are very high compared with HICs, which were reported as 0.1% for mTBI by *BMJ Best Practice*.^22^ Higher mortality in LMICs may partly be attributable to delays in care, driven by both patient-level factors (delayed emergency medical services [EMS] activation, limited awareness, or cultural barriers) and system-level constraints (inadequate EMS, poor referral, and long distances to care).^23,24^ After arrival at health care facilities, absent or malfunctioning CT scanners and the lack of trained staff and neurosurgical facilities further delay definitive care.^25,26,27,28^ Most widely used clinical TBI guidelines, including those from the Brain Trauma Foundation, are developed in high-income contexts and rely on resources often unavailable in LMICs, limiting their applicability and implementation. This gap highlights the need for context-specific, resource-stratified TBI management guidelines in LMIC settings.

After following patients over 1 year, we observed that patients reported an improved QoL. At the end of the study, 17.1%, 72.2%, and 92.2% of patients reported no disability in physical or emotional well-being or functional engagement, respectively. This is similar to one Ugandan study,^20^ which reported long-term neurosurgical outcomes of good recovery, moderate disability, or a return to pre-TBI life in 82.3%, 13.2%, and 84.6% of patients, respectively. Another study, also based in Uganda, reported a return to baseline function in 71% of patients after a TBI.^29^

Surgical interventions were performed in 29.2% of our study population and were associated with lower odds of mortality. This finding is biologically plausible; patients with mild TBI undergoing surgery typically harbor focal, surgically correctable lesions, such as epidural hematomas and depressed skull fractures, which carry a more favorable prognosis when promptly treated compared with diffuse axonal injury. Furthermore, surgery is preferentially performed on patients neurologically and hemodynamically stable enough to tolerate anesthesia, introducing an inherent selection toward less critically ill patients. A similar association was reported in Tanzania, where surgical intervention was linked to an 80% reduction in mortality.^30^ In contrast, a study from Malawi found no significant association between surgical intervention and mortality.^31^ In the absence of standardized, context-specific TBI guidelines and resource frameworks for LMICs, and in the presence of substantial city- and region-specific barriers to care, these observed mortality disparities are concerning; they call into question the generalizability of existing guidelines and underscore the need for further setting-specific research and guideline development.

### Strengths and Limitations

We hope that our study’s strengths, including data collection from both public and private sector hospitals, capture of patients across different socioeconomic backgrounds, and long-term follow-up through 12 months after discharge, render a meaningful step toward building contextually grounded evidence that LMICs urgently need to develop appropriate, resource-sensitive TBI guidelines.

Our study also had several limitations. For example, our study data was retrieved from 2 trauma hospitals in the metropolitan city of Karachi, with advanced neurosurgical facilities and neurosurgeons. Further studies are required to analyze outcomes from rural hospitals with greater resource limitations. Additionally, our inclusion criteria captured only admitted patients and excluded those managed and discharged directly from the emergency department. As most patients with mTBI in high-income settings are discharged from the ED without admission, our cohort represents a more severely affected, hospitalized subgroup. The inclusion of patients with complicated mTBI (characterized by significant intracranial pathology and a high rate of surgical intervention) may have overestimated mortality in our cohort, as these patients occupy a clinical gray zone between mild and moderate TBI. Additionally, as surgical intervention encompasses both cranial and noncranial procedures, the association between surgery and lower mortality should be interpreted cautiously given the potential for selection bias.

## Conclusions

In this cohort study of patients initially diagnosed with mTBIs in Karachi, Pakistan, high mortality was observed, with most deaths occurring within a month of hospital discharge. Patients with a GCS score of 13 and 14 had 2.5 times higher mortality compared with those who had a GCS score of 15. Surgical interventions approximately halved mortality odds, and most survivors recovered both emotionally and functionally. However, one-third of patients with a GCS of 13 and 14 reported residual mild to moderate disability in emotional well-being at 12 months. Our findings underscore the need for structured post-discharge follow-up for high-risk patients. Given the observed mortality and impaired QoL, these results also suggest that current mTBI classifications inadequately capture injury severity in LMIC settings, warranting reconsideration of mTBI definitions in these contexts.

## Notes

Supplement 1. eFigure 1. Schematic Diagram of Study Participants eFigure 2. Turnbull NPMLE Cumulative Mortality Curves by GCS (12-Month) eTable 1. Demographic Case Mix by Type of Procedure eTable 2. Distribution of Epidemiological and Clinical Characteristics Among Mild Traumatic Brain Injury (mTBI) Patients With Follow-up and Loss to Follow-Up eTable 3. Sensitivity Analysis: Cumulative Mortality Under Primary, Best-Case, and Worst-Case Lost-to-Follow-Up (LTFU) Scenarios eTable 4. Distribution of Quality-of-Life Domains in mTBI Patients at 1-, 3-, and 6- and 12-Month Follow-Up Stratified by the GCS eTable 5. Functional Independence Measure (FIM) Categorization in mTBI Patients at 1-, 3-, 6-, and 12-Month Follow-Up

Supplement 2. Data Sharing Statement
