# Supplementary material for: Long-Term Mortality and Quality of Life Among Adults With Mild Traumatic Brain Injury
Source: JAMA Netw Open. 2026 Aug 13;9(8):e2629041. doi: 10.1001/jamanetworkopen.2026.29041 (PMC13474037; doi:10.1001/jamanetworkopen.2026.29041)
Supplement: Supplementary file 1 [file jamanetwopen-e2629041-s001.pdf]

Supplemental Online Content

Kumar K, Rahim KA, Ahmad M, et al. Long-term mortality and quality of life among adults with mild traumatic brain injury. *JAMA Netw Open*. 2026;9(8):e2629041. doi:10.1001/jamanetworkopen.2026.29041

eFigure 1. Schematic Diagram of Study Participants

eFigure 2. Turnbull NPMLE Cumulative Mortality Curves by GCS (12-Month)

eTable 1. Demographic Case Mix by Type of Procedure

eTable 2. Distribution of Epidemiological and Clinical Characteristics Among Mild Traumatic Brain Injury (mTBI) Patients With Follow-up and Loss to Follow-Up

eTable 3. Sensitivity Analysis: Cumulative Mortality Under Primary, Best-Case, and Worst-Case Lost-to-Follow-Up (LTFU) Scenarios

eTable 4. Distribution of Quality-of-Life Domains in mTBI Patients at 1-, 3-, and 6- and 12-Month Follow-Up Stratified by the GCS

eTable 5. Functional Independence Measure (FIM) Categorization in mTBI Patients at 1-, 3-, 6-, and 12-Month Follow-Up

This supplemental material has been provided by the authors to give readers additional information about their work.

© 2026 Kumar K et al. *JAMA Network Open*.

eFigure 1. Schematic Diagram of Study Participants

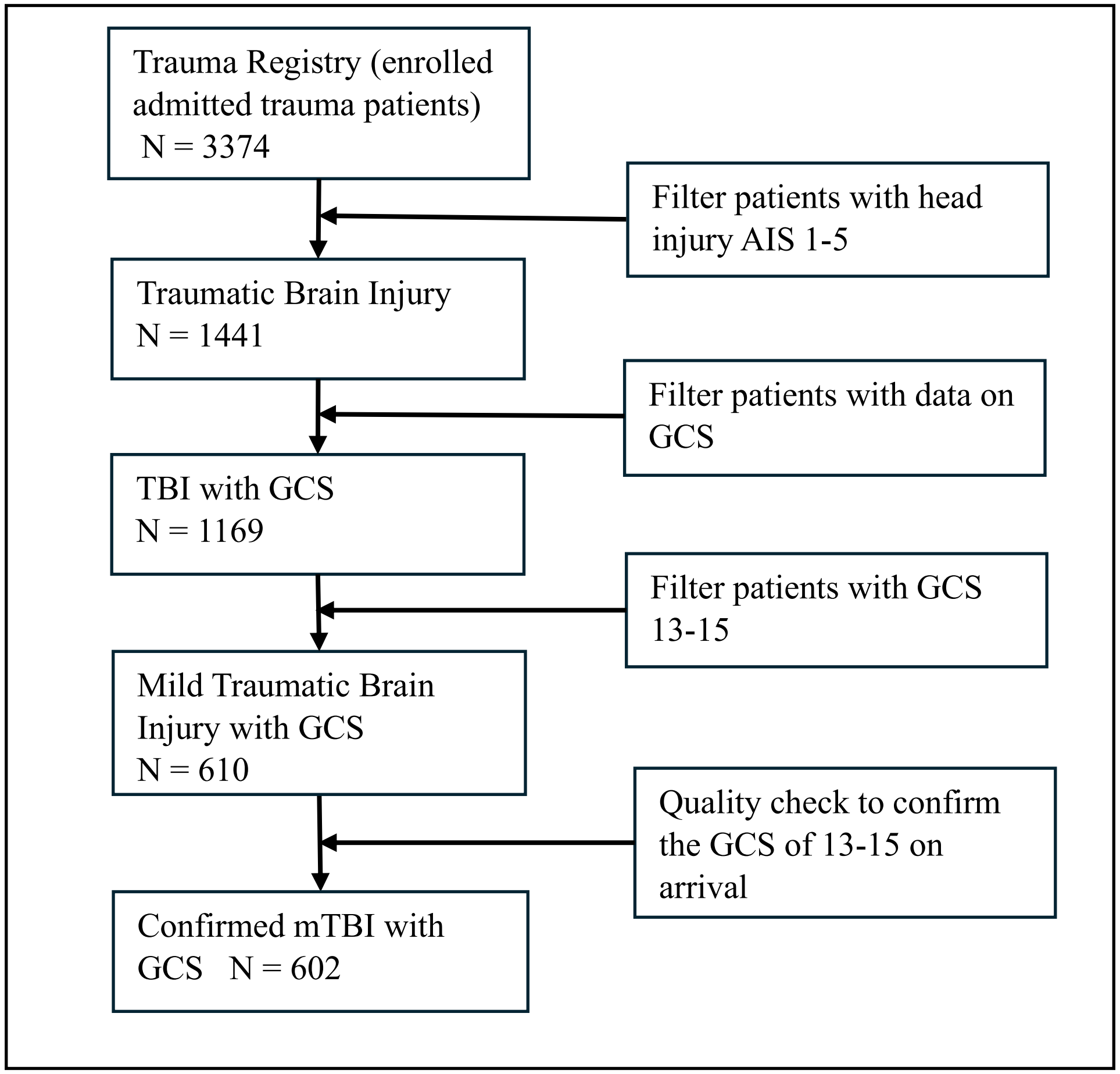

© 2026 Kumar K et al. *JAMA Network Open*.

© 2026 Kumar K et al. *JAMA Network Open*.

eFigure 2. Turnbull NPMLE Cumulative Mortality Curves by GCS (12-Month)

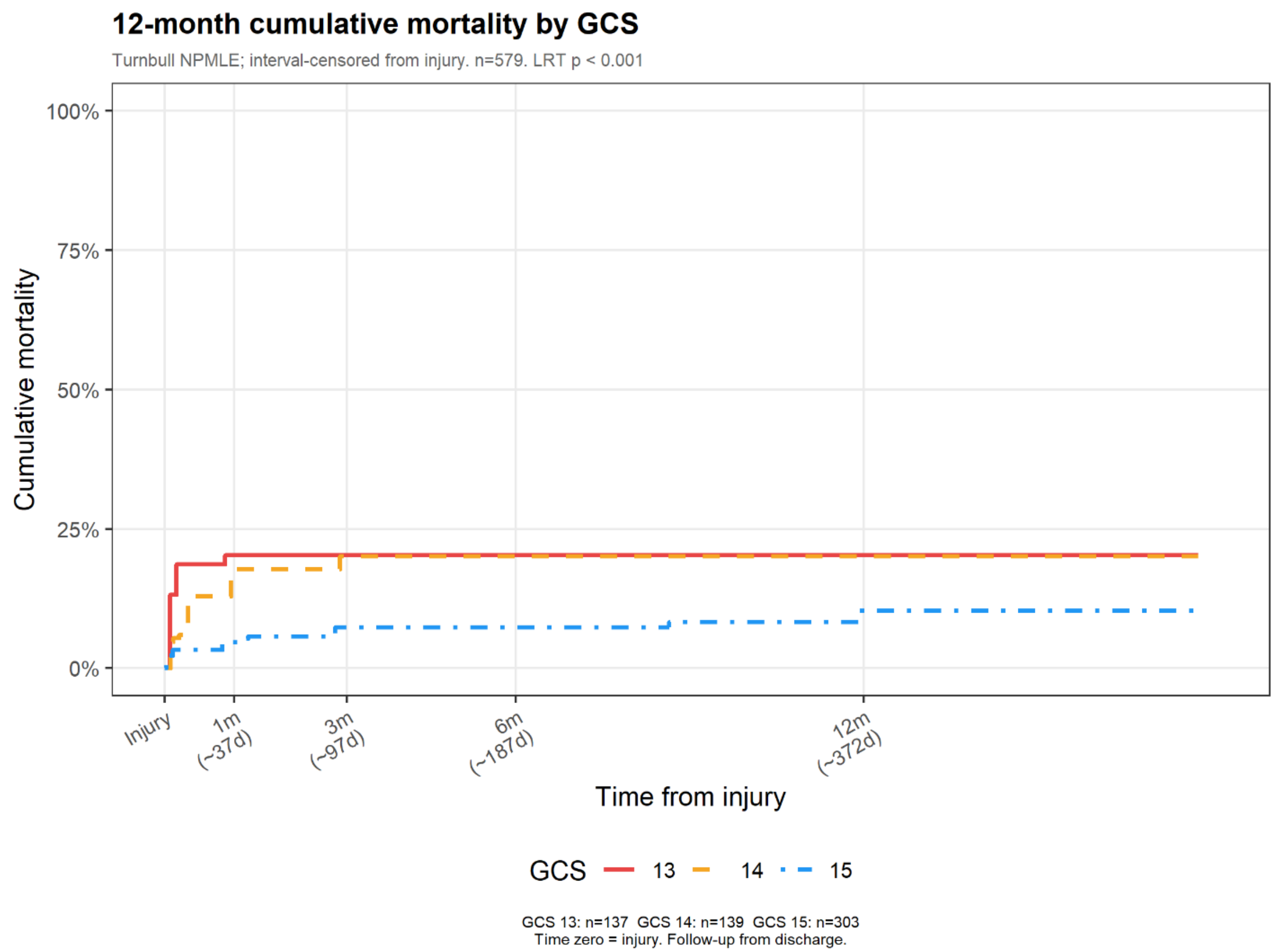

© 2026 Kumar K et al. *JAMA Network Open*.

eTable 1. Demographic Case Mix by Type of Procedure

Characteristics	Craniotomy N = 61	Craniectomy N = 23)	Burrholes N=23
**Age group**			
18-39	47 (77.05)	6 (27.27)	3 (13.04)
40-59	8 (13.11)	8 (36.36)	8 (34.78)
≥ 60	6 (9.84)	8 (36.36)	12 (52.17)
**Gender**			
Male	7 (11.48)	4 (18.18)	3 (13.04)
Female	54 (88.52)	18 (81.82)	20 (86.96)
**GCS**			
13	12 (19.67)	2 (9.09)	3 (13.04)
14	16 (26.23)	6 (27.27)	5 (21.74)
15	33 (54.10)	14 (63.64)	15 (65.22)
**Injury Severity Score (ISS) category**			
Mild (≤8)	3 (4.92)	3 (13.64)	-
Moderate (9-15)	25 (40.98)	3 (13.64)	11 (47.83)
Severe (16-24)	22 (36.07)	14 (63.64)	12 (52.17)
Profound (≥25)	11 (18.03)	2 (9.09)	-
**Cause of Injury**			
RTCs	43 (70.49)	16 (72.73)	8 (34.78)
Fall	14 (22.95)	6 (27.73)	15 (65.22)
Assault	3 (4.92)	-	-
Others	1 (1.64)	-	-
Missing			
**Traumatic Brain Injury (TBI) Diagnosis**			
Contusions	9 (14.75)	6 (26.09)	-
Epidural Hematoma	50 (81.97)	4 (17.39)	1 (4.35)
Subdural Hematoma	6 (9.84)	13 (56.52)	22 (95.65)
Skull fracture	7 (11.48)	1 (4.35)	-
Facial Fracture	5 (8.20)	-	-
Intracerebral Haemorrhage	1 (1.64)	1 (4.35)	-
Pneumocranium	-	-	-
Vertebral Fracture	-	-	-

© 2026 Kumar K et al. *JAMA Network Open*.

© 2026 Kumar K et al. *JAMA Network Open*.

eTable 2. Distribution of Epidemiological and Clinical Characteristics Among Mild Traumatic Brain Injury (mTBI) Patients With Follow-up and Loss to Follow-Up

**Characteristics**	**Follow-up N=421 (%)**	**Loss to follow-up N=181 (%)**	**P-value**
**Age group**			0.07
18-39	223 (52.97)	113 (62.43)	
40-59	128 (30.40)	40 (22.10)	
≥ 60	70 (16.63)	28 (15.47)	
**Gender**			0.6
Male	349 (82.90)	153 (84.53)	
Female	72 (17.10)	28 (15.47)	
**Educational Status**			0.03
No Formal Education	106 (25.54)	64 (37)	
Primary Education	86 (20.72)	40 (23.12)	
Secondary Education	110 (26.51)	34 (19.65)	
Intermediate	52 (12.53)	16 (9.25)	
Graduation and above	61 (14.70)	19 (10.98)	
Unknown	6	8	
**Mechanism of Injury**			0.6
Road Traffic Collisions (RTCs)	302 (71.73)	125 (69.44)	
Fall	92 (21.85)	38 (21.11)	
Assault	17 (4.04)	12 (6.67)	
Others	10 (2.38)	5 (2.78)	
Missing	0	1	
**RTC victim type (RTC = 427)**			0.5
Passenger	38 (12.79)	9 (7.38)	
Pillion passenger	34 (11.45)	13 (10.66)	
Driver	8 (2.69)	4 (3.28)	
Rider	174 (58.59)	75 (61.48)	
Pedestrian	43 (14.48)	21 (17.21)	
Missing	5	3	
**Primary Vehicle (RTC = 427)**			
Motorbike	229 (76.33)	94 (76.42)	
Car	19 (6.33)	6 (4.88)	
Rickshaw	5 (1.67)	3 (2.44	
Bus	5 (1.67)	1 (0.81)	
Pedestrian	33 (11)	17 (13.82)	

© 2026 Kumar K et al. *JAMA Network Open*.

Others	9 (3)	2 (1.63)	
Missing	2	2	
**ISS category**			0.07
Mild (≤8)	42 (9.98)	12 (6.63)	
Moderate (9-15)	217 (51.54)	109 (60.22)	
Severe (16-24)	133 (31.59)	55 (30.39)	
Profound (≥25)	29 (6.89)	5 (2.76)	
**Services**			0.02
High-Dependency Unit (HDU)	333 (80.24)	162 (89.5)	
Intensive-Care Unit (ICU)	9 (2.17)	2 (1.1)	
Ward	73 (17.59)	17 (9.39)	
Missing	6	0	
**Surgical Intervention**			0.4
Yes	127 (30.17)	49 (27.07)	
No	194 (69.83)	132 (72.93)	
**Procedure (n=176)**			0.5
Craniotomy	48 (47.06)	13 (33.33)	
Craniectomy	14 (13.73)	8 (20.51)	
Burr hole Evacuation	15 (14.71)	8 (20.51)	
Elevation of depressed fracture	8 (7.84)	6 (15.38)	
Laceration repair	8 (7.84)	3 (7.69)	
Other	9 (8.82)	1 (2.56)	
Missing	25	10	
**Diagnosis**			
Contusions	143 (33.97)	57 (31.49)	
Epidural Hematoma	132 (31.35)	61 (33.70)	
Subdural Hematoma	111 (26.37)	36 (19.89)	
Skull fracture	68 (16.15)	25 (13.81)	
Facial Fracture	25 (5.94)	8 (4.42)	
Intracerebral Haemorrhage	15 (3.56)	6 (3.31)	
Pneumocranium	12 (2.85)	7 (3.87)	
Vertebral Fracture	11 (2.61)	3 (1.66)	
**GCS**			0.9
13	101 (23.99)	40 (22.1)	
14	100 (23.75)	44 (24.31)	
15	220 (52.26)	97 (53.59)	
**Computed Tomography (CT) scan**			0.6
No	16 (3.73)	5 (2.76)	

© 2026 Kumar K et al. *JAMA Network Open*.

Yes	406 (96.44)	176 (97.24)	
**X-ray**			0.04
No	56 (13.05)	13 (7.18)	
Yes	367 (87.17)	168 (92.82)	
**Magnetic Resonance Imaging (MRI)**			>0.9
No	419 (97.67)	177 (97.79)	
Yes	9 (2.14)	4 (2.21)	
**Length of stay (n = 565)**	2.11 (1.17, 4.23)	1.79 (0.98, 3.04)	0.005
	15	29	

© 2026 Kumar K et al. *JAMA Network Open*.

eTable 3. Sensitivity Analysis: Cumulative Mortality Under Primary, Best-Case, and Worst-Case Lost-to-Follow-Up (LTFU) Scenarios

GCS	Scenario	1 month (%)	3 months (%)	6 months (%)	12 months (%)
Overall	Worst case (LTFU dead)	18.4	23.5	27.5	40.4
Overall	Primary	8.9	12.2	13.1	13.6
Overall	Best case (LTFU alive)	8.3	11.2	12	12.3
13	Worst case (LTFU dead)	29.1	31.8	33.6	44.5
13	Primary	18.6	20.2	20.2	20.2
13	Best case (LTFU alive)	16.8	18.2	18.2	18.2
14	Worst case (LTFU dead)	26.2	31.2	36.2	46
14	Primary	12.9	17.7	20.1	20.1
14	Best case (LTFU alive)	11.4	15.4	17.3	17.3
15	Worst case (LTFU dead)	10.4	16.2	20.9	36
15	Primary	3.3	5.6	7.3	8.3
15	Best case (LTFU alive)	3.1	5.3	6.8	7.5
Best case: LTFU assumed alive throughout. Worst case: LTFU assumed dead at last contact.					

© 2026 Kumar K et al. *JAMA Network Open*.

eTable 4. Distribution of Quality-of-Life Domains in mTBI Patients at 1-, 3-, and 6- and 12-Month Follow-Up Stratified by the GCS

Characteristics	1 Month N = 462 (%)			3 Months			6 Months			12 Months		
	13 N = 96 (%)	14 N = 99 (%)	15 N = 267 (%)	13 N = 96 (%)	14 N = 99 (%)	15 N = 267 (%)	13 N = 96 (%)	14 N = 99 (%)	15 N = 267 (%)	13 N = 96 (%)	14 N = 99 (%)	15 N = 267 (%)
**Physical Well-being and Recovery**												
No disability	16 (17.39)	13 (13.83)	46 (18.18)	31 (34.44)	21 (23.60)	69 (28.28)	19 (20.88)	22 (25.58)	54 (22.50)	12 (15.79)	6 (8.22)	42 (21.43)
Mild disability	29 (31.52)	22 (23.40)	75 (29.64)	27 (30.00)	33 (37.08)	103 (42.21)	41 (45.05)	31 (36.05)	115 (47.92)	47 (61.84)	49 (67.12)	114 (58.16)
Moderate disability	28 (30.43)	35 (37.23)	85 (33.60)	20 (22.22)	27 (30.34)	47 (19.26)	27 (29.67)	26 (30.23)	60 (25.00)	17 (22.37)	14 (19.18)	35 (17.86)
Severe disability	19 (20.65)	24 (25.53)	47 (18.58)	12 (13.33)	8 (8.99)	25 (10.25)	4 (4.40)	7 (8.14)	11 (4.58)	0 (0.00)	4 (5.48)	5 (2.55)
Unknown	4	5	14	6	10	23	5	13	27	20	26	71
**Functional Engagement**												
No disability	63 (68.48)	68 (72.34)	193 (76.28)	74 (82.22)	72 (80.90)	203 (83.20)	80 (87.91)	75 (87.21)	221 (92.08)	72 (94.74)	67 (91.78)	179 (91.33)
Mild disability	6 (6.52)	8 (8.51)	16 (6.32)	6 (6.67)	5 (5.62)	11 (4.51)	9 (9.89)	5 (5.81)	9 (3.75)	2 (2.63)	3 (4.11)	8 (4.08)
Moderate disability	7 (7.61)	5 (5.32)	13 (5.14)	4 (4.44)	3 (3.37)	8 (3.28)	0 (0.00)	2 (2.33)	3 (1.25)	1 (1.32)	0 (0.00)	5 (2.55)
Severe disability	16 (17.39)	13 (13.83)	31 (12.25)	6 (6.67)	9 (10.11)	22 (9.02)	2 (2.20)	4 (4.65)	7 (2.92)	1 (1.32)	3 (4.11)	4 (2.04)
Unknown	4	5	14	6	10	23	5	13	27	20	26	71
**Emotional Well-being**												

© 2026 Kumar K et al. *JAMA Network Open*.

No disability	28 (30.43)	33 (35.11)	128 (50.59)	46 (51.11)	50 (56.18)	151 (61.89)	60 (65.93)	57 (66.28)	185 (77.41)	50 (65.79)	47 (64.38)	152 (77.55)
Mild disability	38 (41.30)	27 (28.72)	71 (28.06)	25 (27.78)	25 (28.09)	56 (22.95)	20 (21.98)	19 (22.09)	37 (15.48)	19 (25.00)	14 (19.18)	34 (17.35)
Moderate disability	14 (15.22)	27 (28.72)	41 (16.21)	13 (14.44)	8 (8.99)	27 (11.07)	6 (6.59)	10 (11.63)	11 (4.60)	7 (9.21)	12 (16.44)	8 (4.08)
Severe disability	12 (13.04)	7 (7.45)	13 (5.14)	6 (6.67)	6 (6.74)	10 (4.10)	5 (5.49)	0 (0.00)	6 (2.51)	0 (0.00)	0 (0.00)	2 (1.02)
Unknown	4	5	14	6	10	23	5	13	28	20	26	71

© 2026 Kumar K et al. *JAMA Network Open*.

eTable 5. Functional Independence Measure (FIM) Categorization in mTBI Patients at 1-, 3-, 6-, and 12-Month Follow-Up

Characteristic	Overall<br>N = 462 (%)	13<br>N = 96 (%)	14<br>N = 99 (%)	15<br>N = 267 (%)
**FIM Category – Admission**				
Complete dependence	35 (7.6%)	17 (17.7%)	9 (9.1%)	9 (3.4%)
Maximal Assistance	105 (22.8%)	43 (44.8%)	25 (25.3%)	37 (13.9%)
Moderate Assistance	96 (20.8%)	25 (26.0%)	35 (35.4%)	36 (13.5%)
Minimal Assistance	107 (23.2%)	9 (9.4%)	22 (22.2%)	76 (28.6%)
Supervision or Setup	81 (17.6%)	2 (2.1%)	4 (4.0%)	75 (28.2%)
Modified Independence	26 (5.6%)	-	4 (4.0%)	22 (8.3%)
Complete Independence	11 (2.4%)	-	-	11 (4.1%)
Missing	1	-	-	1
**FIM Category – Discharge**				
Complete dependence	2 (0.4%)	1 (1.1%)	-	1 (0.4%)
Maximal Assistance	18 (4.0%)	6 (6.5%)	4 (4.1%)	8 (3.1%)
Moderate Assistance	46 (10.2%)	18 (19.4%)	5 (5.2%)	23 (8.9%)
Minimal Assistance	116 (25.8%)	34 (36.6%)	24 (24.7%)	58 (22.4%)
Supervision or Setup	130 (29.0%)	26 (28.0%)	41 (42.3%)	63 (24.3%)
Modified Independence	97 (21.6%)	7 (7.5%)	19 (19.6%)	71 (27.4%)
Complete Independence	40 (8.9%)	1 (1.1%)	4 (4.1%)	35 (13.5%)
Missing	13	3	2	8
**FIM Category – 1 Month**				
Complete dependence	-	-	-	-
Maximal Assistance	6 (1.4%)	1 (1.1%)	2 (2.1%)	3 (1.2%)
Moderate Assistance	13 (3.0%)	2 (2.2%)	4 (4.3%)	7 (2.8%)
Minimal Assistance	19 (4.3%)	3 (3.3%)	3 (3.2%)	13 (5.1%)
Supervision or Setup	41 (9.3%)	15 (16.3%)	9 (9.6%)	17 (6.7%)
Modified Independence	87 (19.8%)	22 (23.9%)	23 (24.5%)	42 (16.6%)
Complete Independence	273 (62.2%)	49 (53.3%)	53 (56.4%)	171 (67.6%)
Missing	23	4	5	14
**FIM Category – 3 Months**				

© 2026 Kumar K et al. *JAMA Network Open*.

Complete dependence	-	-	-	-
Maximal Assistance	1 (0.2%)	1 (1.1%)		
Moderate Assistance	3 (0.7%)		1 (1.1%)	2 (0.8%)
Minimal Assistance	10 (2.4%)	1 (1.1%)	2 (2.3%)	7 (2.9%)
Supervision or Setup	25 (5.9%)	6 (6.7%)	6 (6.8%)	13 (5.3%)
Modified Independence	80 (19.0%)	24 (26.7%)	24 (27.3%)	32 (13.1%)
Complete Independence	303 (71.8%)	58 (64.4%)	55 (62.5%)	190 (77.9%)
Missing	40	6	11	23
**FIM Category – 6 Months**				
Complete dependence	-	-	-	-
Maximal Assistance	-	-	-	-
Moderate Assistance	1 (0.2%)	-	1 (1.2%)	
Minimal Assistance	2 (0.5%)	-	-	2 (0.8%)
Supervision or Setup	7 (1.7%)	-	3 (3.5%)	4 (1.7%)
Modified Independence	72 (17.3%)	17 (18.7%)	21 (24.4%)	34 (14.2%)
Complete Independence	335 (80.3%)	74 (81.3%)	61 (70.9%)	200 (83.3%)
Missing	45	5	13	27
**FIM Category – 12 Months**				
Complete dependence	-	-	-	-
Maximal Assistance	-	-	-	-
Moderate Assistance	1 (0.3%)	-	-	1 (0.5%)
Minimal Assistance	1 (0.3%)	-	1 (1.4%)	-
Supervision or Setup	6 (1.7%)	-	2 (2.7%)	4 (2.0%)
Modified Independence	68 (19.7%)	17 (22.4%)	22 (30.1%)	29 (14.8%)
Complete Independence	269 (78.0%)	59 (77.6%)	48 (65.8%)	162 (82.7%)
Missing	117	20	26	71

© 2026 Kumar K et al. *JAMA Network Open*.
