# Supplementary material for: Long-Term Mortality and Quality of Life Among Adults With Mild Traumatic Brain Injury
Source: JAMA Netw Open. 2026 Aug 13;9(8):e2629041. doi: 10.1001/jamanetworkopen.2026.29041 (PMC13474037; doi:10.1001/jamanetworkopen.2026.29041)
Supplement: Supplementary file 2 [file jamanetwopen-e2629041-s002.pdf]

Data Sharing Statement

Kumar. Long-Term Mortality and Quality of Life Among Adults With Mild Traumatic Brain Injury. *JAMA Netw Open*. Published August 13, 2026. doi:10.1001/jamanetworkopen.2026.29041

Data
Data available: No

Additional Information
Explanation for why data not available: The data analyzed in this study are not publicly available but can be accessed upon request from the corresponding author as per the institutional guidelines.
